# Assessment of Beliefs, Behaviors, and Opinions About Blood Donation in Telangana, India—A Cross Sectional Community-Based Study

**DOI:** 10.3389/fpubh.2021.785568

**Published:** 2021-12-09

**Authors:** Sana Samreen, Ibrahim Sales, Ghada Bawazeer, Syed Wajid, Mansour Adam Mahmoud, Majidah A. Aljohani

**Affiliations:** ^1^Volunteering Researcher in the Clinical Pharmacy Department, College of Pharmacy, King Saud University, Riyadh, Saudi Arabia; ^2^Department of Clinical Pharmacy, College of Pharmacy, King Saud University, Riyadh, Saudi Arabia; ^3^Department of Clinical and Hospital Pharmacy, College of Pharmacy, Taibah University, Al-Madinah, Saudi Arabia; ^4^Pharmaceutical Care Division, King Saud Medical City, Riyadh, Saudi Arabia

**Keywords:** attitudes, motivation, blood donation, Indian adults, fear for needle

## Abstract

**Background and Objectives:** Blood is an essential body fluid primarily required for regulating the body's systems and maintaining homeostasis. In developed and developing countries, concern about the demand and supply for blood is increasing. The current study aims to assess the beliefs, behaviors, and opinions of the public toward blood donation.

**Methods:** This was a cross-sectional study in which a self-created questionnaire with 17-items was used for data collection. The self-administered questionnaire was disseminated between November 2019 and January 2020 through social media (WhatsApp© and Facebook©). Data was analyzed using SPSS program version 26.

**Results:** A total of 356 questionnaires were completed with a response rate of 89%. The majority of participants were male 253 (71.1%), 336 (94.4%) considered blood donation important, 350 (98.3%) believed that blood donation saves lives, and 254 (71.3%) agreed to receive blood from voluntary donors. One-hundred sixty-seven (49.4%) were willing to donate blood voluntarily. The barriers to blood donation were fear of needles 86 (24.2%), fear of contracting a chronic disease 84 (23.6%), and lack of time 40 (11.2%). One day off (91.9%) and receiving a token 73.6% were common motivational factors for blood donation. Overall, 57% of the participants had favorable attitudes toward blood donation and 41.9% were knowledgeable. Favorable attitudes were significantly associated with being married (*P* = 0.018) and having university level of education (*P* = 0.005). Younger participants (18–29 years) had a statistically significant better knowledge than older participants (≥30 years).

**Conclusion:** The respondents displayed positive beliefs, opinions, and motivation toward blood donation. Additionally, most of them considered blood donation an important act and a national duty of every individual and are willing to donate in the future.

## Background

Blood is an essential body fluid primarily required for regulating the body's systems and maintaining homeostasis (1). However, the demand for a safe supply of blood is increasing on a daily basis internationally, and India is no exemption (2, 3). Although previous studies reported that blood transfusions save millions of lives each year, the quality and safety of blood remain a serious concern, particularly in developing countries (2–4). Indeed, concern about the demand and supply for blood is increasing in developed and developing countries (5, 6). However, out of 195 nations, the blood supply of 119 (61%) nations were found inadequate for healthcare needs (5, 7). Interestingly, early findings indicated that India has the world's largest shortage of blood supply. Conversely, the prevalence of blood borne diseases in India is on the rise as blood is essential for the treatment of various diseases (e.g., sickle cell anemia), bleeding disorders (e.g., hemophilia), and cancer. Evidence indicates that India is home to major surgical procedures, such as ~230,000,000 operations, 331,000,000 cancer procedures, and 10,000,000 pregnancy-related operations, every year. Such procedures require a large amount of blood (7, 8).

Blood donation is a pillar of modern medicine and saves millions of lives every year (7, 8). Nevertheless, many hospitalized patients in low- and middle-income countries lack access to safe and free supply of blood in a timely manner. Previous studies estimated that out of the demand for 303 million units of blood worldwide in 2017, only ~272 million units were supplied. In the 119 countries with insufficient blood supply, the shortfall reached 100 million units (7, 8).

Moreover, previous studies conducted in India evaluated the attitudes and motivational factors of the public toward blood donation and reported false beliefs among individuals regarding the effects of blood donation, such as infertility, loss of strength, early aging, and anemia (2, 6). Similarly, studies from developed countries like America and Japan also reported similar barriers toward blood donation (9, 10). However, another study by Shah et al. at a blood bank in a tertiary hospital in Mumbai reported laziness and fear of infection as the major factors for blood donation hesitation (11). In developed countries, published reports demonstrated that lack of access to blood donation centers was the main factor for blood donation hesitation (12–16). Additionally, barriers are different between genders. Failing to meet the eligibility requirements has been reported commonly by females while most males reported that they were never asked to donate blood (17, 18).

According to estimates from the Central Drugs Standard Control Organization (CDSCO), the National Regulatory Authority (NRA) of India, Telangana has 151 blood banks comprised of both private and governmental blood banks, with Hyderabad having the most. Healthy adults between the ages of 18 and 75 who fulfill the donor eligibility requirements can donate blood (19–21). All blood banks are easily accessible to the general public, whether by walk-ins or by appointment (20, 21). Additionally, the city has mobile blood banks making blood donation convenient for all citizens and aims to save lives by connecting donors to blood banks (22).

A dearth of literature exists in this regard, particularly in Telangana, a state in India. Furthermore, international studies on blood donation and its acceptance among the public are limited and evaluating public attitudes and motivations toward blood donation using different methods is required. In addition, the availability of a safe blood supply in healthcare centers is another challenge. Research focused on the attitudes, opinions, and motivations toward blood donation can provide an overall picture of the state of blood supply to healthcare centers not only in India but also across the world. Therefore, the aim of this study was to assess the beliefs, behaviors, and opinions of the general public toward blood donation in Hyderabad, the capital city of Telangana, India.

## Materials and Methods

### Participants and Design

A cross-sectional web-based community study was conducted among adults from November 2019 and January 2020 using a structured, self-administered questionnaire. The study included individuals from Hyderabad city, Telangana state, India who aged more than 18 years, who can read and understand the English language.

### Sample Size Determination

The sample size (*N*) was based on the previous number of blood donors in India (89.5%) (2) and calculated as follows:


$$\begin{array}{l} \text{N}={\text{z}}^{2}\times \text{p}\times \text{q}/{\text{d}}^{2}, \end{array}$$

where *N* is the minimum sample size; *z* denotes the level of confidence according to the normal standard distribution that corresponds to the 95% confidence interval (*z* = 1.96); *p* stands for the prevalence rate of blood donors (0.895); q = (1 – *p*); and d pertains to the desired degree of accuracy or tolerated margin of error (5%; 0.05). Substituting these values into the equation, the following equation is derived:


$$\begin{array}{l} \text{N}={\left(1.96\right)}^{2}\times 0.90\times \left(1-0.90\right)/{\left(0.05\right)}^{2}=356. \end{array}$$

Therefore, *N* = 356.

### Questionnaire Design

A structured, self-administered questionnaire in the English language was prepared through an extensive literature review (12–15). The questionnaire was composed of the following demographics: age, gender, level of education, and employment status. The second part was intended to collect data on the attitudes, opinions, and motivations toward blood donation using 17 items with binary answers and multiple-choice questions. The questionnaire was assessed for the level of comprehensiveness, clarity, avoidance of ambiguity, and content validity by two senior researchers and one clinical pharmacy professor who were experts in the field. A pilot study was conducted on 10 randomly selected individuals who did not mention any suggestions or corrections related to the wording, length, and format of the questionnaire. The pilot results were excluded from the main findings. The reliability of the final questionnaire was assessed using internal consistency. The Cronbach's alpha value was 0.71, indicating an acceptable reliability of each item.

### Data Collection

The final questionnaire Google Forms^®^ link was sent to the participants through WhatsApp© and Facebook© prefaced by the eligibility requirements of participation such as consent and age restrictions. The snowball technique was used to collect data, that is, one participant was requested to refer other individuals to participate. An invitation link containing the questionnaire was sent randomly to the participants without previous measures. First, the research team targeted friends and family members, explained the objective of the study through phone calls and messages, and invited them to fill and forward the questionnaires to family, friends, acquaintances, and any other eligible individual currently living in Telangana. Complete responses to the survey were considered written informed consent as the survey included a statement on consent. To facilitate completion, the respondents were sent frequent reminders about the importance of their participation and requested to submit the completed questionnaire.

### Data Extraction

**Figure 1:**
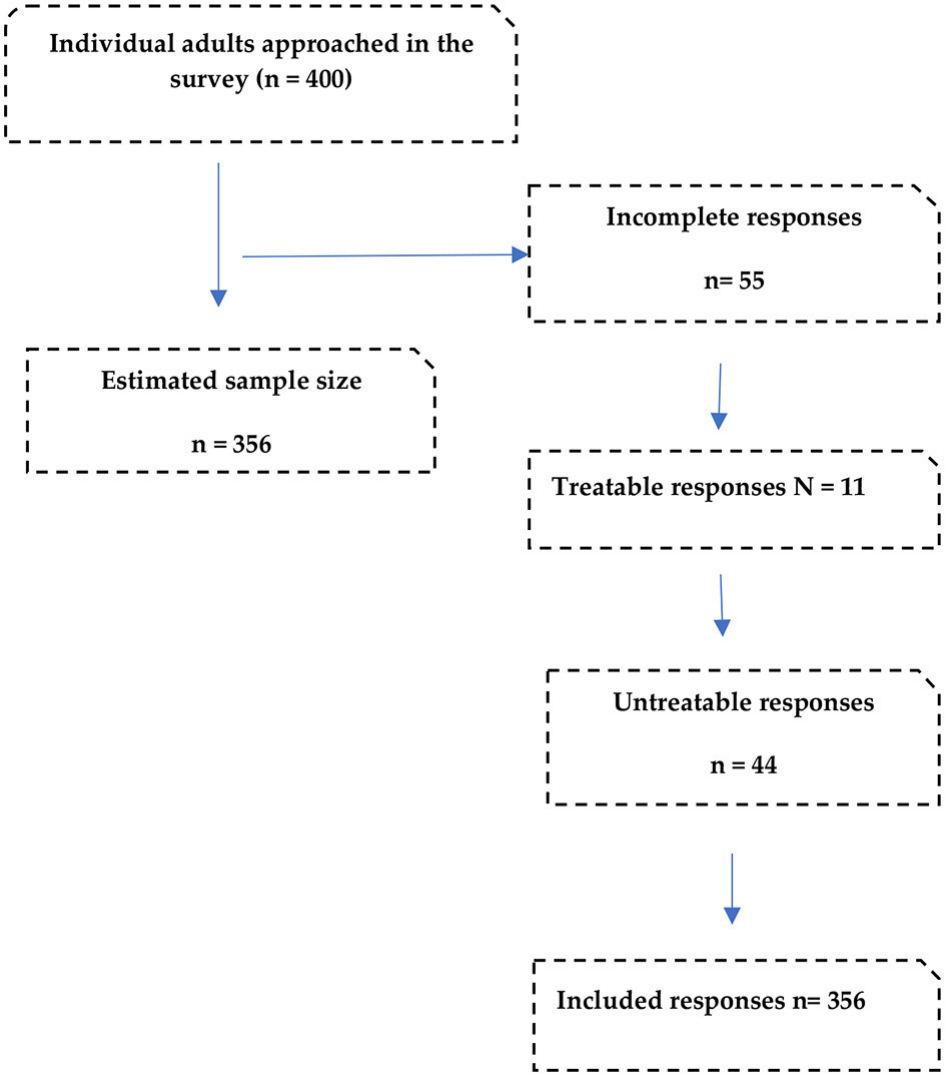
Flowchart of responses.

The submitted questionnaires were checked for accuracy and completeness. Missing or incomplete responses were excluded (Figure 1).

### Data Analysis

Data were further extracted to exclude bias in sample selection, which was limited to only the central region, and analyzed using Microsoft Excel followed by a descriptive analysis. Categorical data were calculated as frequencies and percentages. The Statistical Package for the Social Sciences version 22.0 (SPSS Inc., Chicago, IL, USA) was used for statistical analysis. A chi-square test was used to identify associations between variables. A difference with a *P*-value of >0.5 was considered statistically significant.

## Results

### Participant Demographics

A total of 400 questionnaires were returned, out of which 44 (9%) were incomplete and thus excluded. Therefore, the final number of respondents was 356 for a response rate of 89%. The gender-wise greater proportion of the respondents were male 253 (71.1%), and the majority 291 (81.7%) were aged between 18 and 29 years. Of the 356 respondents, 233 (65.4%) were single, and slightly more than half 186 (52.2%) were unemployed. Most of the study participants, 282 (79.9%), were University graduates. Table 1 provides the demographics of the respondents.

**Table 1:** Demographics of the participants (*n* = 356).

**Characteristics**	**Description**	**Frequency**	**Percentages (%)**
Age in years	18–29 years	291	81.7
	>30 years	65	18.3
Gender	Male	253	71.1
	Female	103	28.9
Marital status	Married	123	34.6
	Single	233	65.4
Level of education	High school	71	20.1
	University	282	79.9
Employment status	Employed	158	44.6
	Students	10	2.8
	Unemployed	186	52.2

### Attitudes Toward Blood Donation

The majority of respondents 336 (94.4%) recognized the importance of blood donation. Nearly all the participants 350 (98.3%) stated that blood donation can help save lives, whereas 254 (71.3%) agreed to receive blood from voluntary donors. In terms of blood donation being a national duty, 203 (57%) agreed to the statement. However, slightly less than half of the respondents 167 (46.9%) would voluntarily donate blood if needed, whereas 171 (48%) may donate blood to friends and families in the future. Table 2 provides detailed information of the respondents' attitudes.

**Table 2:** Attitudes and opinions toward blood donation (*n* = 356).

**Statements**	**Frequency**	**Percentage (%)**	**Favorable attitude (%)**
Do you think that blood donation is an important act?			
Yes	336	94.4	94.4
No	20	5.6	
Do you think donating blood can save lives?			
Yes	350	98.3	98.3
No	6	1.7	
Do you accept blood donation from others (volunteers)?			
Yes	254	71.3	71.3
No	102	28.7	
Will you donate the blood in the future?			
Yes	341	95.8	95.8
No	15	4.2	
Do you think blood donation is a national duty?			
Yes	203	57	57
No	153	43	
What will be your reason for donating?			
As a volunteer	167	49.4	
For my family and friends	171	50.6	
**Attitude score**			
Unfavorable attitude (score of 4 or less out of 6)	153	43	
Favorable attitude (scored of 5–6 out of 6)	203	57	

### Barriers Toward Blood Donation

**Figure 2:**
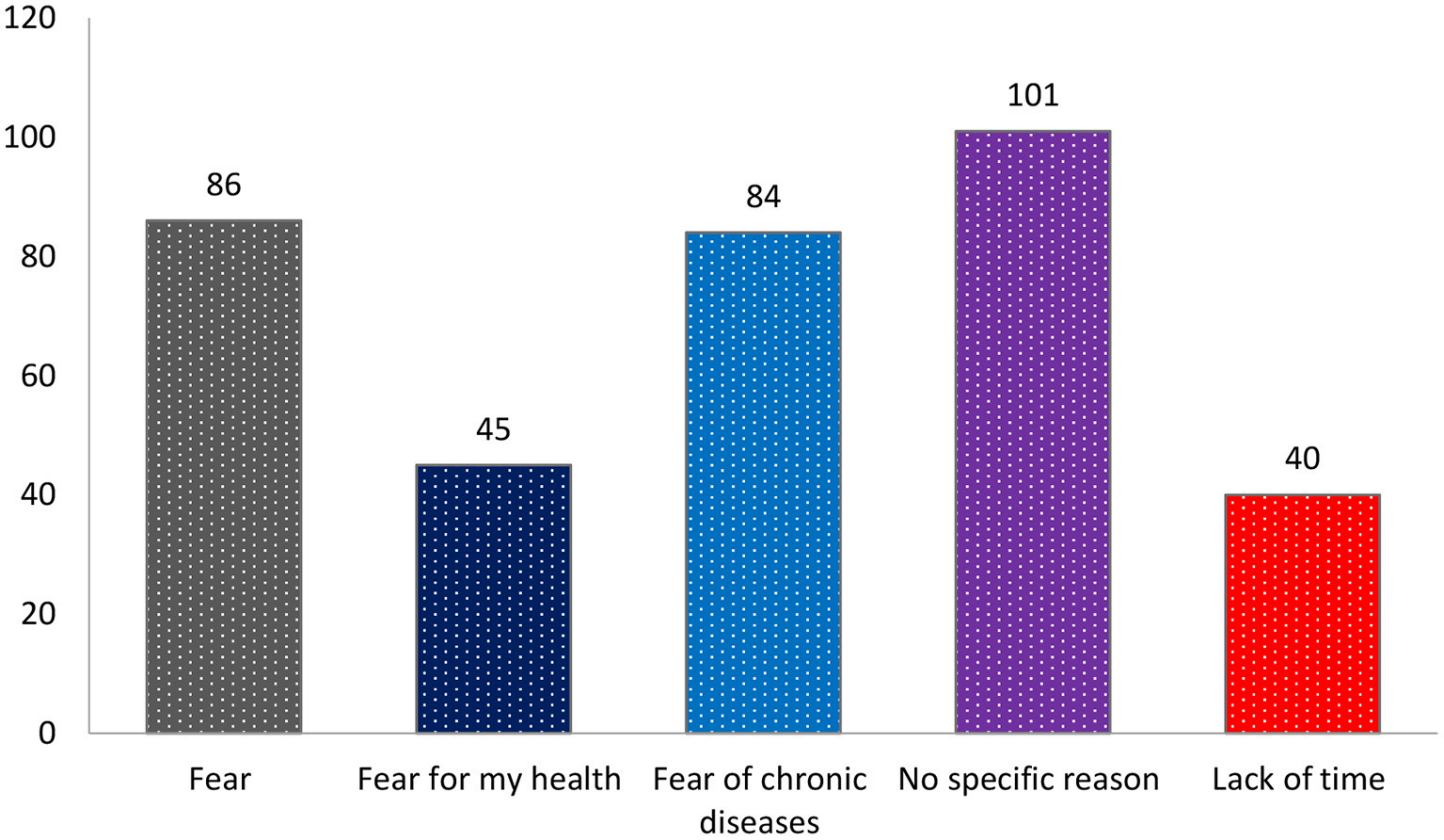
Fears and misconceptions preventing donors from donating blood.

Figure 2 describes the fears and misconceptions that prevent individuals from donating blood. Approximately one-fourth of the respondents 86 (24.2%) avoid donation for fear of needles, 101 (29%) reported no specific reasons, whereas 84 (23.6%) reported a fear of contracting chronic diseases. However, only 40 (11.2%) reported lack of time as the main barrier to blood donation (Figure 2).

### Knowledge and Motivational Factors

Approximately 49.7% of the respondents reported that a healthy person may donate once a year, whereas 14 and 11.3% answered twice and thrice a year, respectively. The majority (73.8%) preferred to donate in blood banks, whereas 83.1% of the subjects were not rejected for blood donation in the past year. Regarding motivational factors, the majority (91.9%) agreed that a 1-day leave should be provided as compensation, whereas 73.6 and 25% preferred that a token and money should be given as rewards, respectively. Furthermore, the majority (93%) agreed that blood donation is an important valuable act and reported satisfaction in donating blood as a means of helping friends and family members. Tables 3, 4 provide detailed descriptions of the responses.

**Table 3:** Knowledge and motivations about blood donation (*n* = 356).

**Variables**	**Frequency**	**Percentage (%)**	**Correct answer %**
How many times can a person donate blood per year?			7.4
One time a year	167	49.7	
Two times a year	47	14	
Three times a year	38	11.3	
Four times in a year	42	12.5	
Five times in a year	17	5.1	
**Six times a in a year**	25	7.4	
If you agree to donate the blood, where do you prefer to donate?			73.8
**Blood bank**	256	73.8	
Residence	48	13.8	
Workplace	43	12.4	
According to your knowledge, can people with any blood type donate blood?			78.7
**Yes**	280	78.7	
No	10	2.8	
I don't know	66	18.5	
Do you agree that donors should be paid to promote blood donation?			75
Yes	89	25	
**No**	267	75	
Knowledge categories			
Knowledgeable (scores of <3 out of 4)	149	41.9	
Not knowledgeable (scores of 3 or 4 out of 4)	207	58.1	

**Table 4:** Factors motivating blood donation.

**Variables**	**Frequency**	**Percentage (%)**
Do family and friends consider blood donation as an important and valuable act and encourage you to donate?		
Yes	322	90.4
No	34	9.6
Would you donate blood if given a leave from work?		
Yes	327	91.9
No	29	8.1
Does donating blood make you feel like you have helped your family members or friends?		
Yes	331	93
No	25	7.0
Do you agree that a token should be given to donors as a motivational factor?		
Yes	262	73.6
No	94	26.4

Married individuals had a statistically significant favorable attitude compared to single participants (*P* = 0.018). Participants with a university level of education had statistically significant favorable attitudes compared to those with high school education (*P* = 0.005) (Table 5).

**Table 5:** Cross-tabulation between demographic characteristics and attitudes categories.

	**Number of respondents**	**Unfavorable attitude (*N* = 153)**	**Favorable attitude (*N* = 203)**	***P*-value**
Age				0.489
18–29 years	Respondents	128	163	
	% within age	44.0%	56.0%	
	% within attitudes categories	83.7%	80.3%	
≥30 years	Respondents	25	40	
	% within age	38.5%	61.5%	
	% within attitudes categories	16.3%	19.7%	
Gender				0.906
Male	Respondents	108	145	
	% within gender	42.7%	57.3%	
	% within attitude categories	70.6%	71.4%	
Female	Respondents	45	58	
	% within gender	43.7%	56.3%	
	% within attitude categories	29.4%	28.6%	
Marital status				0.018
Married	Respondents	42	81	
	% within marital status	34.1%	65.9%	
	% within attitude categories	27.5%	39.9%	
Single	Respondents	111	122	
	% within marital status	47.6%	52.4%	
	% within attitude categories	72.5%	60.1%	
Educational status				0.005
University	Respondents	133	149	
	% within educational level	47.2%	52.8%	
	% within attitude categories	86.9%	74.5%	
High school	Respondents	20	51	
	% within educational level	28.2%	71.8%	
	% within attitude categories	13.1%	25.5%	
Employment status				0.239
Employed	Respondents	61	97	
	% within employment status	38.6%	61.4%	
	% within attitudes categories	40.4%	47.8%	
Unemployed	Respondents	87	99	
	% within employment status	46.8%	53.2%	
	% within attitude categories	57.6%	48.8%	
Student	Respondents	3	7	
	% within employment status	30.0%	70.0%	
	% within attitude categories	2.0%	3.4%	

*Fishers exact test and chi-square test*.

*Younger participants (18–29 years) had a statistically significant better knowledge than older participants (≥30 years). A statistically significant differences in knowledge categories were also reported among marital status (P = 0.003), educational status (P = 0.001) and employment status (P < 0.001)*.

Younger participants (18–29 years) had a statistically significant better knowledge than older participants (≥30 years). A statistically significant differences in knowledge categories were also reported among marital status (*P* = 0.003), educational status (*P* = 0.001) and employment status (*P* < 0.001) (Table 6).

**Table 6:** Cross-tabulation between demographic characteristics and knowledge categories.

	**Number of respondents**	**Not knowledgeable (*N* = 207)**	**Knowledgeable (*N* = 147)**	***P*-value**
Age				<0.001
18–29 years	Respondents	153	138	
	% within age	52.6%	47.4%	
	% within knowledge categories	73.9%	92.6%	
≥30 years	Respondents	54	11	
	% within age	83.1%	16.9%	
	% within knowledge categories	26.1%	7.4%	
Gender				0.479
Male	Respondents	144	109	
	% within gender	56.9%	43.1%	
	% within knowledge categories	69.6%	73.2%	
Female	Respondents	63	40	
	% within gender	61.2%	38.8%	
	% within knowledge categories	30.4%	26.8%	
Marital status				0.003
Married	Respondents	85	38	
	% within marital status	69.1%	30.9%	
	% within knowledge categories	41.1%	25.5%	
Single	Respondents	122	111	
	% within marital status	52.4%	47.6%	
	% within knowledge categories	58.9%	74.5%	
Educational status				0.001
University	Respondents	177	105	
	% within educational level	62.8%	37.2%	
	% within knowledge categories	85.5%	71.9%	
High school	Respondents	30	41	
	% within educational level	42.3%	57.7%	
	% within knowledge categories	14.5%	28.1%	
Employment status				<0.001
Employed	Respondents	111	47	
	% within employment status	70.3%	29.7%	
	% within knowledge categories	53.6%	32.0%	
Unemployed	Respondents	86	100	
	% within employment status	46.2%	53.8%	
	% within knowledge categories	41.5%	68.0%	
Student	Respondents	10	0	
	% within employment status	100.0%	0.0%	
	% within knowledge categories	4.8%	0.0%	

*Fishers exact test and chi-square test*.

## Discussion

The respondents indicated positive beliefs, behaviors, and opinions toward blood donation. Furthermore, the majority agreed that blood donation is an important act and helps save lives. Additionally, nearly all respondents indicated willingness to donate if asked, and ~47% would donate blood as volunteers. The current results are better than those of previous studies conducted in the capital of India, where the authors reported that 69% of the respondents displayed positive attitudes toward blood donation and considered blood donation as the duty of every individual to the community (23). Joshi and Meakin conducted a study among Indian non-donors living in England and reported a variety of attitudes, but generally positive ones (4). Olaiya surveyed citizens in Nigeria, a developing country, and reported that 92.9% of the participants donated blood and demonstrated positive attitudes toward blood donation (24). However, Majdabadi et al. reported moderate attitudes among medical students in Tehran (6). Additionally, other previous studies proposed that increased awareness and motivational factors were associated with good knowledge and attitudes toward blood donation (6, 12, 24–26).

Previously published studies indicated an association between good knowledge, attitudes, and opinions toward blood donation and the availability and safe supply of blood in transfusion centers (12–15). This objective can be achieved further through increased awareness about blood donation and its importance and in-depth research on the motivational factors that encourage donors to donate. The present study found that one's fear of needles, fear of contracting chronic diseases, and lack of time were potential barriers that limit blood donation among the respondents. However, Shah et al. argued that fear of infection (21%), fear of needles (15%), and laziness were the major factors (46%) for the blood donation hesitancy (11). Similarly, a recent population-based study by Alfouzan et al. pointed to lack of time (45%) and access to blood donation centers (41.3%) (12). Karim et al. reported fear among Bangladeshi population (26). Interestingly, Dubey et al. reported that respondents were not requested to donate blood, which was considered the main potential barrier among respondents (2). Moreover, Abdurrahman and Saleh identified the side effects of receiving blood or blood components, health problems, fear of blood, medical errors, time restraints, lack of required conditions for donation, and fear of acquiring infections (e.g., HIV) as barriers among donors (13). The different types of fear among subjects, as reported by the present and previous studies, should be addressed by highlighting the importance of blood donation through programs that promote awareness, whereas misconceptions regarding infections due to blood donation should be elucidated through various educational programs about donation.

The current study demonstrated that the respondents held positive attitudes, opinions, and motivations toward blood donation in India. The findings were consistent with those of previous studies conducted in developed and developing countries and found overall positive and attitudes and perceptions toward blood donation (2, 11–13). In the current study, however, the majority of participants were willing to donate blood if asked and nearly half would donate as volunteers. These findings supported Dubey et al., who stated that most potential donors (57.25%) would donate only if required for family or friends with self-sacrifice as a lesser priority (16%) (2). However, Abdurrahman and Saleh reported that 61.2% of participants revealed that their main goal for donation is helping others and saving lives, even individuals they do not know (13). Additionally, Dubey et al. found that 13.5% of the respondents were non-donors, where 7.75% agreed to donate to gain awareness about their HIV status (2). Lastly, non-monetary incentives, if carefully targeted, can attract and retain donors (2, 15).

In the current study, the majority of respondents suggested a token, leave from work, and cash money as motivational incentives for donating blood. The results were comparable to those of Alfouzan et al. who reported 1 day off (81.4%), tokens (31.5%), and money (18.9%) as motivating factors (12). Similarly, Baseer et al. surveyed university students and identified that saving lives (98.4%), serving humanity (96.9%), and helping family and friends (95.3%) were the main motivations for donation (14). Karim et al. reported that family background, physical status, urgency for family, awareness/knowledge, and maturity level were the factors that increased participant willingness to donate (26). Irrespective of gender and age, individuals hold personal beliefs and misconceptions about donating and accepting blood anonymously (2, 13). However, the respondents of the present study agreed with importing blood from abroad. Additionally, previous studies proposed that socio-demographic, organizational, physiological, and psychological factors may influence the decision of individuals to donate and accept blood (13–16). Many studies reported that blood donation is a religious duty, whereas the current results revealed that blood donation is a national duty (13–16). An encouraging fact observed in the study is that individuals are motivated to donate blood.

In this study being younger, educated, married, and employed were shown to have superior knowledge, whereas married individuals and those with higher education had more favorable attitudes scores. Melku et al. revealed similar findings in Ethiopia, with younger people being found to have more knowledge (27). Similarly, according to Javaeed et al., being a female gender was found to have a high level of understanding about blood donation (28). In Bangladesh, Karim et al. found that a parent's education was substantially related to the study participants' blood donation behaviors (26). This data revealed that the participants' education and age were important determinants in their blood donation decisions.

As such, creating awareness in the community and shedding light on the misconceptions about blood donation can aid healthcare facilities and the public in ensuring the availability of blood when needed. Such outcomes demand the need for additional educational and awareness programs specific to blood donation in Asian communities. In addition, this study proposes that increased knowledge about blood donation through education and awareness campaigns may encourage and motivate the general community and subsequently establish a sufficient supply of viable blood based on voluntarism, which is essential to the healthcare setting.

This study has strengths that should be mentioned. It is one of the first studies to be conducted in Telangana's capital, Hyderabad, which has the most blood donation centers. Secondly, it explored the motivations, perceptions, barriers, and possible strategies to mitigate blood donation hesitancy in one of the world's largest developing countries.

This study has some limitations. First, the study is limited to Hyderabad and included a small sample size; therefore, the findings are not generalizable to the entire state of Telangana. Second, the findings are reliant on the completion of the questionnaire, which may generate false answers and introduce the possibility of bias. Additionally, the study did not assess a full scope of motivational factors for blood donation (such as religion, socioeconomic status), also, the study did not collect past experiences with blood donation or transfusion, as participants who previously donated or received blood may have different attitude and knowledge compared to naïve donors. Lastly, most of the participants are young, and college educated, hence extrapolation to older generation and people with less than university education is limited. Given these limitations, the study suggests that future research should employ a larger sample size from various regions throughout India and reaching wider socioeconomic classes with a greater focus on the opinions, attitudes, and motivations as well as barriers toward blood donation. Future questionnaires should include additional motivational factors for donation and perceptions from historical donors.

## Conclusion

The findings provide insight into the beliefs, behaviors, opinions, and motivations that are likely to encourage blood donation in Hyderabad. Importantly, the findings represent positive attitudes and motivation toward blood donation, which can provide reference for healthcare systems and blood banks in improving their supply and for supporting the development of programs that aim to ensure a sufficient supply of viable blood in a timely manner. Therefore, education programs that promote motivation and ensure a safe and healthy supply of blood should be advocated at both the national and global levels.

## Data Availability Statement

The raw data supporting the conclusions of this article will be made available by the authors, without undue reservation.

## Ethics Statement

Ethical review and approval was not required for the study on human participants in accordance with the local legislation and institutional requirements. The patients/participants provided their written informed consent to participate in this study.

## Author Contributions

SW: conceptualization, data curation, formal analysis, and visualization. SS: writing—original draft preparation and review and editing. IS: funding acquisition and writing—review and editing. MM: formal analysis and writing—review and editing. GB and MA: writing—review and editing. All authors have read and agreed to the published version of the manuscript.

## Conflict of Interest

The authors declare that the research was conducted in the absence of any commercial or financial relationships that could be construed as a potential conflict of interest.

## Publisher's Note

All claims expressed in this article are solely those of the authors and do not necessarily represent those of their affiliated organizations, or those of the publisher, the editors and the reviewers. Any product that may be evaluated in this article, or claim that may be made by its manufacturer, is not guaranteed or endorsed by the publisher.
